# Comparative Material and Mechanical Properties among Cicada Mouthparts: Cuticle Enhanced with Inorganic Elements Facilitates Piercing through Woody Stems for Feeding

**DOI:** 10.3390/biology12020207

**Published:** 2023-01-29

**Authors:** Kristen E. Reiter, Cynthia Perkovich, Katelynne N. Smith, Jiansheng Feng, Gene Kritsky, Matthew S. Lehnert

**Affiliations:** 1 Department of Biological Sciences, Kent State University at Stark, North Canton, OH 44720, USA; 2 Biology and Toxicology Department, Ashland University, Ashland, OH 44805, USA; 3 School of Polymer Science and Polymer Engineering, University of Akron, Akron, OH 44325, USA; 4 Department of Biology, Mount St. Joseph University, Cincinnati, OH 45233, USA

**Keywords:** Hemiptera, insect cuticle, energy dispersive X-ray spectroscopy, nanoindentation, cicada feeding

## Abstract

**Simple Summary:**

Cicadas are one of the most popular insects. Their loud mating songs, newsworthy mass emergences and prolonged lifespan underground (17 years in some species) make cicadas a model organism for building bridges between scientific studies and the public. A key aspect of cicada biology is that the adults use their tube-like mouthparts to pierce through the hard wood of trees to feed on fluids, an ability that suggests that their mouthparts might have adaptations for piercing wood, such as increased hardness and stiffness. Here, we aimed to determine if the cuticle that comprises cicada mouthparts is enhanced with metals and other inorganic elements that could increase cuticular hardness and stiffness. We used scanning electron microscopy and energy dispersive X-ray spectroscopy to study mouthpart morphology and to determine which elements are found in the mouthpart cuticle. We found metals and other inorganic elements in the cicada mouthparts. Additionally, nanoindentation was also used to determine mouthpart mechanical properties. Metals were mostly located at the tip of the mouthparts (the part that pierces wood) and were harder than other regions. These findings are not only valuable to the fields of material sciences, coevolution, and ecology, but provide another interesting aspect of cicada biology.

**Abstract:**

Adult cicadas pierce woody stems with their mouthparts to feed on xylem, suggesting the presence of cuticular adaptations that could increase hardness and elastic modulus. We tested the following hypotheses: (a) the mouthpart cuticle includes inorganic elements, which augment the mechanical properties; (b) these elements are abundant in specific mouthpart structures and regions responsible for piercing wood; (c) there are correlations among elements, which could provide insights into patterns of element colocalization. We used scanning electron microscopy (SEM) and energy dispersive X-ray spectroscopy (EDS) to investigate mouthpart morphology and quantify the elemental composition of the cuticle among four cicada species, including periodical cicadas (*Magicicada* sp.). Nanoindentation was used to quantify hardness and elastic modulus of the mandibles. We found 12 inorganic elements, including colocalized manganese and zinc in the distal regions of the mandible, the structure most responsible for piercing through wood; nanoindentation determined that these regions were also significantly harder and had higher elastic modulus than other regions. Manganese and zinc abundance relates to increased hardness and stiffness as in the cuticle of other invertebrates; however, this is one of the first reports of cuticular metals among insects with piercing-sucking mouthparts (>100,000 described species). The present investigation provides insight into the feeding mechanism of cicadas, an important but understudied component of their life traits.

## 1. Introduction

The expansive diversity of insect feeding habits is considered an important contributor to their massive ecological and evolutionary successes [1,2,3]. Although several insect lineages have retained the ancestral structural ground plan of chewing mouthparts [4], many insect groups have evolved an array of mouthpart shapes, chemistries and structural organizations that facilitate access to new food sources and feeding habits [5,6,7]. In addition, some insects have mouthparts augmented with inorganic elements, including transition metals, which influence the mechanical properties of the cuticle by hardening structures, increasing resistance to wear and affecting elastic modulus (i.e., Young’s modulus) [8,9,10].

Metals and other inorganic elements have been reported in the mouthparts of a wide array of invertebrate groups, including flies (Diptera) [11], ants (Hymenoptera) [8,12], beetles (Coleoptera) [13,14], termites (Blattodea) [9,15,16], grasshoppers (Orthoptera) [17], polychaete worms (Phyllodocida) [18] and spiders (Araneae) [19,20], among others. Of the true bugs (Hemiptera, 80,000+ described species), which have piercing-sucking mouthparts [21,22,23], only the Western conifer seed bug, *Leptoglossus occidentalis*, a seed piercer, has been reported to have manganese in its mouthpart stylets [24].

Several hemipteran species are important pests that pierce and feed on fruits and other crops [25,26,27], which could require metal-augmented mouthparts for piercing, thus warranting further study in this insect group. A noteworthy group of hemipterans that might have mouthparts modified with inorganic elements are cicadas (Cicadidae, 3000+ described species). Cicadas produce the loudest sounds among insects (over 150 dB in *Cyclochila australasiae*) [28] and the 13- and 17-year periodical cicadas (*Magicicada* sp.) are famous for their mass emergences in eastern North America, contributing to their popularity among scientists and the public [29,30]. In addition, trees of various species acquire damage during mass emergences of periodical cicadas due to the females using their ovipositors to pierce through woody stems to lay their eggs (i.e., flagging) [31,32,33]. For this reason, we previously investigated the material properties of the ovipositor cuticle and found a variety of inorganic elements at the distal ovipositor tip, the region responsible for piercing through wood [34].

Many insects pierce wood for oviposition (e.g., some Hymenoptera) [35,36]. However, cicadas are unique in that they also use their mouthparts to pierce wood for xylem feeding, i.e., it is uncommon for an organism to have two separate cuticular tools needed for piercing through wood for separate purposes. The immature cicadas feed primarily on the roots of grasses and small lateral tree roots [37], which might be softer due to the moisture content in the soil [38], but adults pierce through the harder stems and branches [39]. The piercing-sucking mouthparts of cicadas consist of a stylet comprised of two medial maxillae, which form a salivary duct and a food canal, and two lateral mandibles that in some cicada species have bumps at the distal region [39]. The stylet is enclosed by a sheath-like labium that allows the stylet to project distally into wood when feeding.

Here, we explore the material composition and mechanical properties of cicada mouthparts and hypothesize that the cuticle has inorganic elements concentrated in mouthpart regions responsible for piercing wood. In addition, studies have shown some correlative traits between inorganic elements present in the cuticle [40,41], which might work synergistically for specific mechanical adaptations. Here, we tested the following predictions:

1. The presence and accumulation of inorganic elements will vary based on the mouthpart structure, the location on the structure and by cicada species.
2. Mouthpart regions with transition metals will be harder and have higher elastic modulus.
3. Correlations between elements might exist, showing patterns of colocalization.

## 2. Materials and Methods

### 2.1. Species

Pinned specimens of four cicada species were obtained from the insect collection at Mount St. Joseph University (Cincinnati, OH, USA): the annual cicada, *Neotibicen linnei* (Smith and Grossback, 1907) (n = 5), the 17-year periodical cicadas, *Magicicada cassinii* (Fisher, 1852) (n = 5), *M. septendecim* L. (n = 5) and *M. septendecula* (Alexander and Moore, 1962) (n = 3). Only females were used for each species to remove the possibility of sexual dimorphisms that could impact our analysis.

### 2.2. Mouthpart Morphology

Cicada mouthparts were imaged with scanning electron microscopy (SEM). The labial sheath was removed from the head by sliding it distally with forceps so that the stylet (mandibles and maxillae) remained attached to the head. The heads, with attached stylets and the detached sheaths were then placed through an ethanol dehydration series (15 min each in 70%, 80%, 90% and 100% EtOH) followed by at least 24 h in hexamethyldisilazane. The mandibles and maxillae were removed from the head and individually secured to an aluminum stub using carbon graphite tape so that the dorsal side was exposed. The labial sheath was positioned similarly. The mouthparts were sputtercoated with 10 nm of platinum using an EMS 150TS sputter coater and imaged at 75× magnification for the sheaths and 300× magnification for the mandibles and maxillae using a JEOL 6010LV SEM.

**Figure 1:**
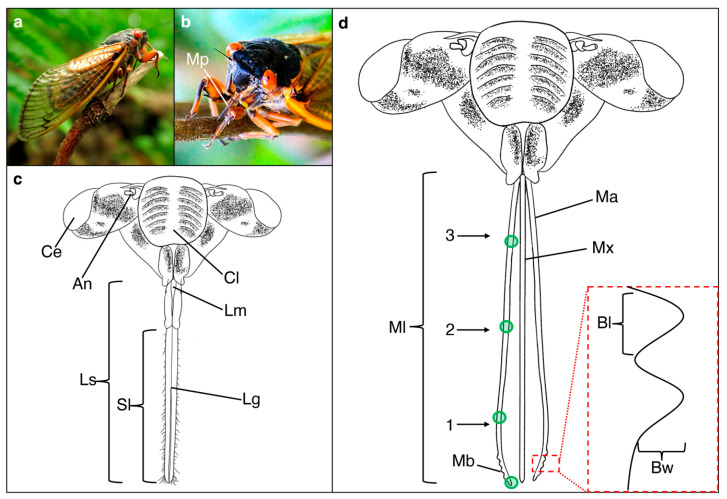
Cicada mouthpart structures and locations for measurements. (**a**) Digital image of the lateral view of a periodical cicada (*Magicicada* sp.) on a twig. (**b**) Digital image of a *Magicicada* sp. using its mouthparts (Mp) to pierce into a tree stem. (**c**) Illustration of anterior view of cicada head showing the labial sheath (Ls) that has a longitudinal groove (Lg) and the compound eyes (Ce), antennae (An) and clypeus (Cl). Only the distal section of the sheath was used to acquire measurements, such as sheath length (Sl) and widths. (**d**) Illustration of the anterior view of a cicada with the sheath removed, exposing the mandibles (Ma) and maxillae (Mx). The mandible and maxilla length (Ml) were measured along with the widths at the distal (1), middle (2) and proximal (3) regions. The mandibular bumps (Mb) at the distal tip of the mandible were counted and bump length (Bl) and width (Bw) were measured (shown in inset). Elemental composition was determined at a proximal, middle, distal and tip region for all mouthpart structures (locations shown with green circles on the mandible). Digital image shown in (**a**) was acquired from Wikipedia.com (accessed on 22 December 2022) under the creative comment attribution 2.0 license and image (**b**) was acquired by James DeMers and uploaded as a free to use image from Pixabay.com (accessed on 22 December 2022).

Serial images were combined into single composite images in Microsoft PowerPoint and measurements of structures were acquired using ImageJ software [42]. Mandible and maxilla lengths were measured from the base to the distal tip for each individual. Only the distal segment of the labial sheath had its length measured because this section remained intact during the removal process. Each mandible, maxilla and distal segment of the sheath had its width measured in three locations along the length: a distal (Location 1), middle (Location 2) and proximal location (Location 3) (Figure 1). The locations for width measurements were determined by first drawing a line along the length for each structure on the serial image in Microsoft PowerPoint, then dividing that line into three equal-sized parts to represent different regions. The middle of each region was used as one of the three width measurements per structure. The distal region of a mandible was further examined to determine bump number, bump length and bump width (Figure 1). Bump length was measured at the base and bump width was measured from the base to its distal tip. For individuals with multiple bumps, only the middle bump was measured.

### 2.3. Elemental Composition with Energy-Dispersive X-ray Spectroscopy

Energy-dispersive X-ray spectroscopy (EDS) (X-Max50, Oxford Instruments) was used to quantify the elemental composition of mouthpart cuticle. The mandible, maxilla and sheath were analyzed (20 kV, spot size 60–65, magnifications >200×) for three minutes at four specified locations: a proximal location at 25% of the mouthpart length from the base, a middle location at 50%, a distal location at 75% and a distal tip location (Figure 1). The locations were identified using SEM and the magnification was increased until the entire field of view consisted only of the location of interest (i.e., no background or debris visible). EDS data was reported in Aztec software (Oxford) as percentage weight for each detected element. EDS is capable of identifying and measuring the elemental composition for most elements that have an atomic number higher than that of Neon (atomic number of 10).

### 2.4. Hardness and Elastic Modulus Measurements

Mandibles from individuals of *N. linnei*, *M. cassinii* and *M. septendecula* (n = 3 individuals for each species) were removed with a razorblade and placed into a droplet of dH_2_O on a glass slide. We did not study the hardness and elastic modulus of *M. septendecim* due to a lack of available specimens. Forceps were used to manipulate the mandibles while a paintbrush wetted with dH_2_O was used to remove debris. The mouthparts were then positioned on a dry glass slide so that the lateral side was exposed and secured with clear tape. A Bruker Hysitron TI Premier nanoindenter with a Ti-0045 cono-spherical probe (90° cone angle, 5 µm tip radius, diamond coated) was used with Triboscan v9.6 software to acquire hardness and elastic modulus measurements. It was determined prior to experimentation that the actual shape of the indenter probe agreed with the default area-function of a spherical probe, −*A* = *π*·*h*^2^ + 2*πR*·*h*, where A is the cross-sectional area, h is the indentation depth and R is the probe tip radius. Three measurements were acquired on the lateral side of each mandible at the proximal and distal locations, each with load-controlled quasi-static indentation tests using a standard trapezoidal loading function (5 s loading, then 2 s dwell, followed by 5 s unloading times) and a maximum load set to 1000 N. Force-displacement curves were analyzed by the TriboScan software, which is based on the Oliver-Pharr Method [43] and allowed obtaining of the reduced modulus and hardness values. The reduced modulus was converted to elastic modulus using the assumption that the Poisson’s ratio of the cicada mouthparts is 0.3 [44,45].

### 2.5. Statistics

Assumptions were tested a priori of statistical analysis. Necessary transformations were performed on variables that violated assumptions of independence, normality, homoscedasticity or multicollinearity. An analysis of variance (ANOVA) was used to determine if there were significant differences (*p* < 0.05) in mouthpart morphology among and within species with JMP v 16 statistical software. Significant differences in means were ranked using a post-hoc Tukey HSD test.

All statistical analyses for the EDS measurements were performed in R software [46]. To increase statistical power and minimize a Type I error associated with multiple dependent variables, a MANOVA was used to understand the relationship between the abundance of elements (organic and inorganic) in the mouthparts (mandible, maxilla and sheath), locations on each mouthpart (tip, distal, mid and proximal) and species (*M. cassinii*, *M. septendecim*, *M. septendecula* and *N. linnei*). Using the dplyr package in R [47], the model included organic and inorganic elements as the response variables, mouthpart structure and location within the mouthpart as fixed effects and species were included as random effects. Variables that were statistically significant (*p* < 0.05) were further investigated using ANOVAs and post-hoc Tukey HSD tests to analyze differences between means. To investigate patterns within individual species, additional ANOVA models were created for each species with mouthpart and location within the mouthpart as fixed effects and elements as the response variables. Carbon and oxygen were excluded from species models because of their prominence in all species.

Pearson’s correlations were run between elements to determine correlative effects in the following groups: mouthpart structure and cicada species. Correlations between elements were used to determine if elements colocalized with other elements within specific mouthparts and mouthpart structures. We also performed principal component analyses (PCAs) and created plots using the ggfortify package in R [48] to determine patterns of inorganic elements abundance in mouthpart structures and locations within structures. The PCA models used a correlation matrix that standardizes each variable to better explain structure and variable relationships [49]. Carbon and oxygen were excluded from the analyses.

A linear discriminant analysis was used to determine if mouthpart morphology or EDS data (inorganic elements only) can be used as an accurate species classification system. A hierarchical clustering analysis (Ward’s method with standardized data) was used to simultaneously evaluate the morphology and EDS inorganic-element data to produce a dendrogram in order to determine if the four cicada species show phylogenetic grouping patterns.

## 3. Results

### 3.1. Mouthpart Morphology

#### 3.1.1. Differences in Lengths and Widths of Mouthpart Structures among Species

There were significant differences in the lengths of each mouthpart structure among species (Supplementary Tables S1 and S2). The mandible and maxilla were significantly longer in *N. linnei* than the *Magicicada* sp. (*p* < 0.0001 for both structures). There also were differences in the sheath length among species with *N. linnei* having the longest sheath and significantly shorter sheaths for *M. septendecim* and *M. cassinii* (*p* = 0.0002) (Supplementary Tables S1 and S2).

The sheath at the distal region (Location 1) was significantly wider for *N. linnei* than *M. septendecula* and *M. cassinii* (*p* = 0.0016) and sheath width for *M. septendecim* was the largest for the periodical cicadas. The sheath width at Location 2 and Location 3 had similar patterns where it was widest in *N. linnei* (*p* = 0.0001, *p* = 0.0002, respectively). The maxilla width had a similar pattern among species and was widest in *N. linnei* (Location 1, *p* < 0.0001; Location 2, *p* = 0.0017; Location 3, *p* < 0.0001). All locations along the mandible were generally wider in *N. linnei* than the periodical cicada species (*p* = 0.0511) and Locations 2 and 3 were significantly wider in *N. linnei* (*p* = 0.0009, *p* = 0.0246, respectively) (Supplementary Tables S1 and S2).

The extent of tapering of each mouthpart was determined by comparing the widths at each location within species (Supplementary Tables S1 and S3). The mandible, maxilla and sheath widths were similar along the length of each structure in *M. cassinii* (*p* = 0.6640, *p* = 0.2612, *p* = 0.2204, respectively), indicating a lack of extensive tapering. For *M. septendecim*, the maxilla and sheath widths were consistent along its length (*p* = 0.1100, *p* = 0.1004, respectively), but the mandible width significantly tapered along its length (*p* = 0.0338). For *M. septendecula*, the mandible and sheath widths were similar along their lengths (*p* = 0.4763, *p* = 0.8065, respectively), but the maxilla significantly tapered distally (*p* = 0.0046). The mandible width of *N. linnei* was consistent along its length (*p* = 0.4801), but there was significant tapering in the maxilla and the sheath (*p* = 0.0076, *p* = 0.0094, respectively) (Supplementary Tables S1 and S3).

#### 3.1.2. Tip Morphology of Mandibles and Maxillae

**Figure 2:**
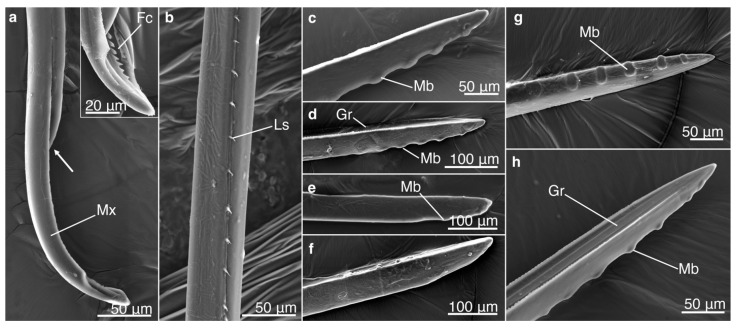
Scanning electron microscopy (SEM) images of maxilla and mandible morphology of different cicada species. (**a**) Lateral view of the maxilla (Mx) of *Magicicada cassinii* showing the sliding mechanism (the arrow indicates the retracted maxilla) that allows the food canal to be exposed. The insert shows the exposed distal tip of the maxilla of *M. septendecula* with the food canal (Fc). (**b**) Proximal region of the maxilla of *M. cassinii* with the linking structures (Ls) that keep the two halves assembled during sliding movements. The distal region of the mandibles of *M. septendecim* (**c**), *M. septendecula* (**d**) and *M. cassinii* (**e**) has bumps (Mb); however, these were mostly lacking in this individual of *Neotibicen linnei* (**f**). Each mandible has a medial groove (Gr), shown in (**d**) and (**h**), that partly encloses the maxilla. (**g**,**h**) Lateral and medial views, respectively, of the mandible of *M. septendecula*.

All observed maxillae consisted of two sections that together created a salivary duct and a food canal. In addition, the maxillae appeared to have the ability to perform a sliding mechanism where a maxilla can move posteriorly, exposing the food canal. Linking structures were observed on the proximal locations, which likely keep the two parts together while performing the sliding mechanism (Figure 2). Bumps were located near the tip of the mandible (Figure 2); however, the number of bumps significantly differed among species (*p* = 0.0019) with approximately six bumps for *M. septendecim* and *M. septendecula* and two bumps for *M. cassinii* and *N. linnei* (Supplementary Tables S4 and S5). The mean width and length of the bumps were similar among species (*p* = 0.4162, *p* = 0.1301, respectively).

### 3.2. Inorganic Elements by Mouthpart Structure, Location and Cicada Species

The mouthpart structures (sheath, mandible and maxilla), locations on the structures, species and the interactions of these variables significantly affected the abundance of inorganic elements (*p* < 0.05, Supplementary Table S6).

#### 3.2.1. Organic Nonmetals: Carbon, Oxygen

Carbon (C) and oxygen (O) made up the largest percentage of elements among all mouthpart structures, location on structures, species and the interactions of these variables (*p* > 0.05). When species were analyzed as a random effect, C was not statistically different among the mouthpart structures or locations on the structures (*p* > 0.05, Supplementary Table S7).

#### 3.2.2. Alkali Metal, Halogens and Non-Metals: Chlorine, Potassium, Sodium, Sulfur, Phosphorus

**Figure 3:**
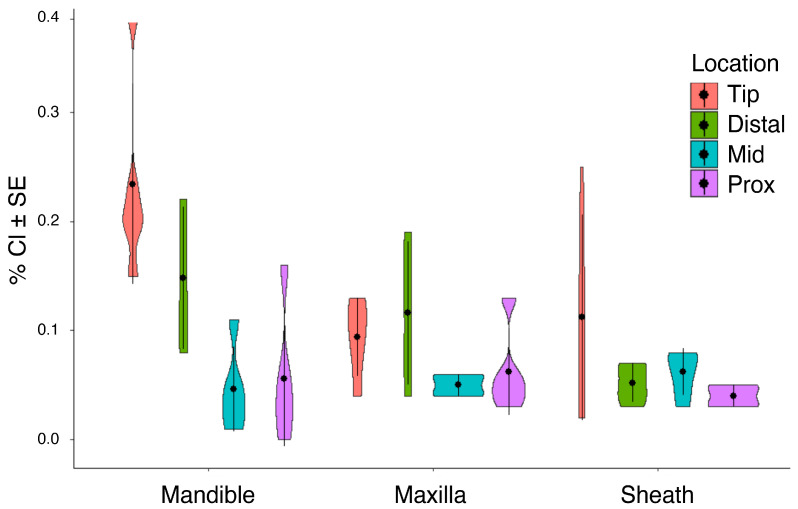
Violin plots of chlorine (Cl) abundance in *Magicicada cassinii* mouthpart structures and locations within structures. Center points in each violin represent the mean, with standard error bars.

Potassium (K) and chlorine (Cl) did not change overall in cicada mouthparts (Supplementary Table S7). When mouthpart locations were compared per structure, however, there was a trend of higher concentrations of Cl near the distal regions, particularly for the mandible (Figure 3). When individual species were analyzed per structure, *Magicicada* sp. had less variation in Cl abundance than *N. linnei* (Table 1).

Only *M. cassinii* had consistent changes of K and Cl (Table 1); K abundance decreased from the tip to the proximal base of each mouthpart structure (Supplementary Figure S1) and the mandibles showed a similar trend to Cl (Table 1). Sodium (Na) was significantly different among mouthpart structures, locations within mouthpart structures, species and their interactions (mouthpart structure, *p* = 0.001; location within structure, *p* = 0.009; species, *p* < 0.001, Supplementary Figure S2, for interactions see Supplementary Table S7). There were greater amounts of Na in *M. septendecula*, particularly in the sheath, compared to other *Magicicada* sp. (all *p* < 0.05). In the mandibles of *M. cassini* and *M. septendecim*, Na was more abundant at the tip and distal regions compared to other structures (all *p* < 0.05, Supplementary Figure S2). Despite having overall more Na than other species, there were no differences of Na abundance in mouthpart structures and locations within mouthparts among *M. septendecula* specimens (Supplementary Figure S2, Table 1). Sulfur (S) and phosphorus (P) remained consistent across mouthpart structures and locations of mouthparts (all *p* > 0.05, Table 1, Supplementary Table S7).

#### 3.2.3. Alkaline Earth Metals: Calcium, Magnesium

With species as a random effect, calcium (Ca) remained constant across mouthpart structures and locations on structures (all *p* > 0.05, Supplementary Table S7). Magnesium (Mg) significantly varied among species (*p* = 0.016) and with species as a random factor, Mg was detected in greater amounts in the sheath compared to other structures (*p* = 0.008, *p* = 0.028, respectively) and had significantly higher abundances in the distal regions (all *p* < 0.05). Only *N. linnei* and *M. septendecim* showed differences in Mg abundance (Table 1). Interactions between mouthpart structures and locations showed a significantly greater concentration of Mg in the distal region of the sheath compared to other structures and locations (*p* = 0.032, Supplementary Figure S3).

#### 3.2.4. Transition, Post-Transition Metals and Metalloids: Aluminum, Iron, Manganese, Silicon, Zinc

**Figure 4:**
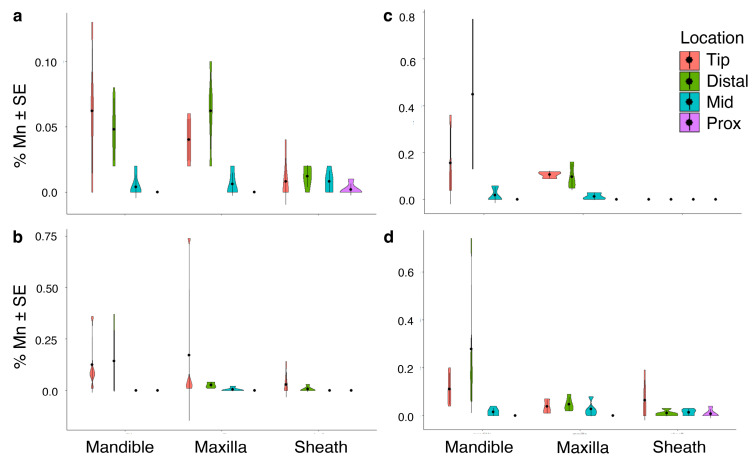
Violin plots showing abundances of manganese (Mn) at different locations of cicada mouthpart structures. (**a**) *Magicicada cassinii,* (**b**) *M. septendecim,* (**c**) *M. septendecula and* (**d**) *Neotibicen linnei*. Center points in each violin represent the mean, with standard error bars.

**Figure 5:**
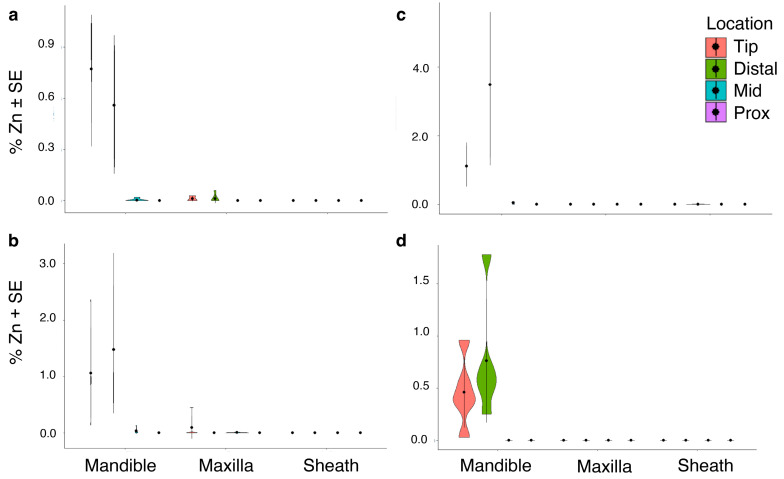
Violin plots demonstrating differences in zinc (Zn) abundance at different locations of cicada mouthpart structures. (**a**) *Magicicada cassinii,* (**b**) *M. septendecim,* (**c**) *M. septendecula and* (**d**) *Neotibicen linnei*. Center points in each violin represent the mean, with standard error bars.

Aluminum (Al) was not detected in individuals of *M. septendecula* (Table 1). In fact, Al accumulation was only statistically significant in *M. septendecim* (Supplementary Figure S4, Table 1) and was most abundant in the distal and tip regions (*p* < 0.001). There were no significant differences in iron (Fe) or silicon (Si) abundance across mouthpart structures, locations on structures, or species (all *p* > 0.05, Table 1). Fe was not detected in *M. septendecim* or *M. septendecula* specimens (Table 1). There were significant differences in manganese (Mn) and zinc (Zn) abundances in mouthpart structures and locations within the mouthparts (manganese, *p* < 0.001, Figure 4; zinc, *p* < 0.001, Figure 5), with a higher percentage composition of Mn and Zn at the tip and distal regions of the mandibles of *M. septendecula* and *M. cassini* (all *p* < 0.05).

#### 3.2.5. Correlations between Organic and Inorganic Elements by Mouthpart Structure

O was negatively associated with several inorganic elements (Table 2); however, the inorganic elements varied with mouthpart structure. Na and Zn, for example, were negatively associated with O in mandibles (Table 2). In the maxillae, O was negatively associated with S, Cl, K and Ca (Table 2) and in the sheath O was negatively associated with Na, S, Cl and K (Table 2). Cl was positively associated with K, Na and Ca in all mouthpart structures (Table 2).

#### 3.2.6. Correlations between Organic and Inorganic Elements by Species

Individuals of *M. cassinii* had strong negative correlations between Si and organic elements, except for P (Supplementary Table S8). Individuals of *M. cassinii* and *M. septendecim* both showed strong positive relationships between Cl and several inorganic elements, including Na, Mn and Zn (Supplementary Tables S8 and S9), but *M. septendecula* did not (Supplementary Table S10). C and O in *M. septendecim* showed a strong negative relationship between several inorganic elements including S, Cl and K. In *M. septendecim*, S had strong positive correlations with Cl, K, Mn and Ca (Supplementary Table S9). In *M. septendecula*, there were strong negative correlations between C and O and alkaline metals (Cl, K and Mn, see Supplementary Table S10). Similar to *M. septendecim, N. linnei* demonstrated negative correlations between O and Cl, K and S (Supplementary Table S11). Aside from these correlations, *N. linnei* showed different patterns of associations between elements from the *Magicicada* species; for example, *N. linnei* was the only species that did not show associations (positive or negative) between carbon and other elements. Individuals of *N. linnei* also showed several positive relationships between Al and Ca, Cl and S (Supplementary Table S11), which were lacking in the *Magicicada* sp.

#### 3.2.7. Generalized Patterns of Metal Bioaccumulation in Cicada Mouthparts and Locations on the Mouthparts

**Figure 6:**
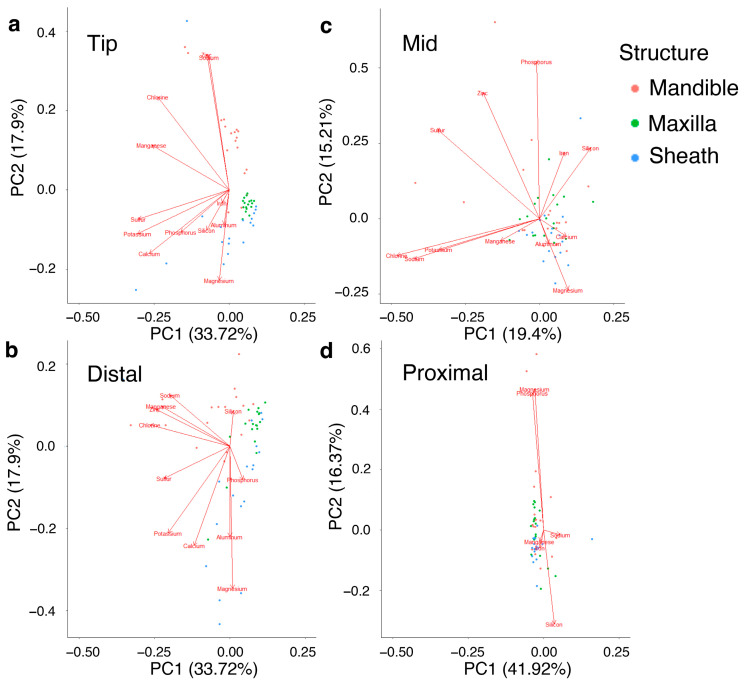
Principal component analysis (PCA) of the location of inorganic elements in the mouthparts of cicadas. Locations are in order from the tip to the proximal base, including (**a**) tip, (**b**) distal, (**c**) mid, and (**d**) proximal. Individual mouthparts are represented by different colors in the ordination.

For individual mouthpart structures, PC1 explained 43% of the variation in the mandibles, 44% in maxillae and 32% in the sheath (Supplementary Figure S5 and Table S12). PC2 explained 16% in mandibles, 17% in maxillae and 23% in sheath (Supplementary Table S12). K and Cl explained much of the variation in PC1 for all three mouthpart structures and Ca also was important for understanding PC1 for the maxillae and sheath (Supplementary Figure S5 and Table S13). As previously stated, Al was not present in the mandibles or the sheath but explained much of the variation in PC1 of the maxillae. For locations on the mouthpart structures, PC1 explained 34% in the tip location, 36% in the distal location, 19% in the mid location and 42% of the variation in the proximal location (Figure 6, Supplementary Table S14). In the mandibles, Si explained most variation in PC2 for the distal location and Mg explained most of the variation in the distal region of the sheath (Figure 6, Supplementary Table S15).

### 3.3. Patterns in Cicada Grouping by Morphological Measurements and EDS Results

A linear discriminant analysis revealed an accurate classification system when only morphological measurements were used (100% accurate classification for each species) (Table 3). However, when only EDS measurements were used, the classification system inaccurately grouped several individuals. For example, only 60% of individuals of *M. cassinii* were correctly classified, with 40% inaccurately classified as *M. septendecim* or *N. linnei*. For *M. septendecim* and *N. linnei*, only 60% were accurately classified within each species group. In contrast, 100% of *M. septendecim* were correctly classified (Table 3).

**Figure 7:**
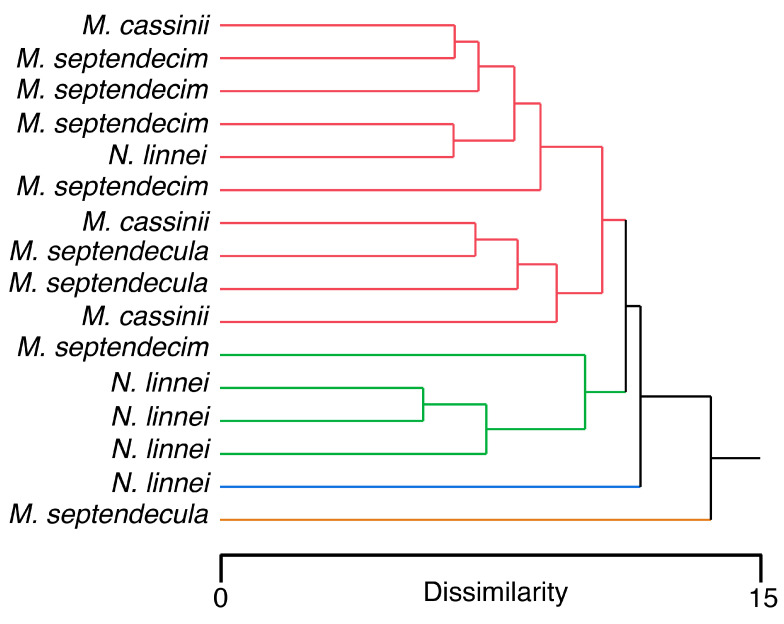
Dendrogram showing patterns of similarities among cicada individuals based on EDS and morphological measurements. The different colors represent the four clusters that were assigned to the hierarchical cluster analysis to represent the four species of cicadas.

EDS and morphological measurements were combined for a hierarchical cluster analysis (four clusters to represent each species). The resulting dendrogram showed a generally inaccurate classification system (Figure 7). For example, one cluster consisted of one individual of *M. septendecula* and another entire cluster had only one individual of *N. linnei*. A third cluster showed relatively accurate classification with three individuals of *N. linnei*, but also grouped an individual of *M. septendecim*. The last cluster consisted of a mixed assembly of all four species.

### 3.4. Hardness and Elastic Modulus

**Figure 8:**
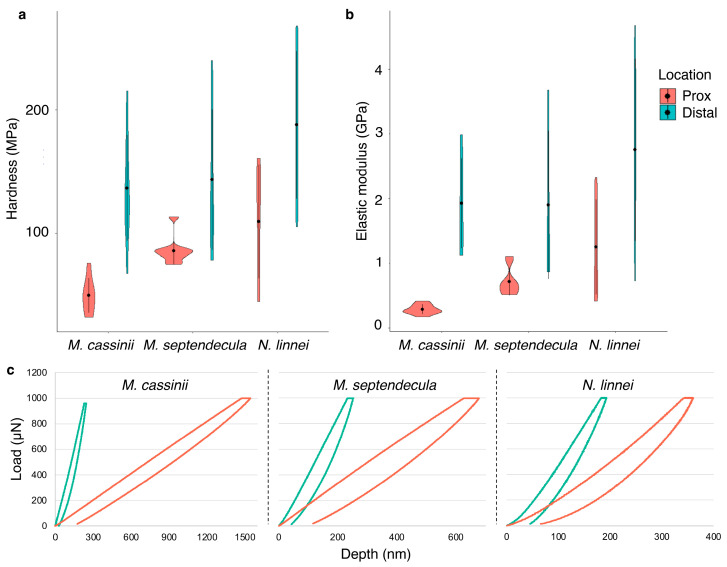
Hardness and elastic modulus measurements with force curves of cicada mandibles. (**a**) Hardness and (**b**) elastic modulus values were significantly greater in the distal regions than proximal regions. (**c**) Representative force curves of cicada species as determined by nanoindentation with a maximum force of 1000 N.

The mechanical properties of cicada mandibles were determined by quantifying the elastic modulus and hardness at the distal and proximal locations using nanoindentation. There were significant differences between the proximal and distal locations for both measurements for all species. For *M. cassinii*, elastic modulus and hardness were greater in the distal region (both *p* < 0.0001) (Figure 8). Similar patterns were observed in *M. septendecula* (elastic modulus, *p* = 0.0077; hardness, *p* = 0.0091) and *N. linnei* (elastic modulus, *p* = 0.0141; hardness, *p* = 0.0065) (Supplementary Table S16). Comparisons of each location among species revealed a pattern where there were significant differences in proximal locations, but not the distal locations (elastic modulus, *p* = 0.2706; hardness, *p* = 0.1252) (Supplementary Table S17). The elastic modulus in the proximal location was significantly higher for *N. linnei* than the *Magicicada* sp. (*p* = 0.0005) and *N. linnei* and *M. septendecula* had significantly harder proximal regions than *M. cassinii* (*p* = 0.0007) (Figure 8).

## 4. Discussion

The present study is the first to reveal the elemental composition of the mouthpart cuticle of cicadas and to our knowledge, the first to find a wide array of inorganic elements in piercing-sucking mouthparts of insects (100,000+ species). Insects exhibit a range of mouthpart types but piercing-sucking mouthparts are found in the true bugs (Hemiptera), thrips (Thysanoptera), lice (Psocodea), some flies (Diptera), fleas (Siphonaptera) and some moths (Lepidoptera) [6]. The composition of the mouthpart cuticle of these groups remains relatively unstudied. Here, we found that cicada mouthparts contain transition metals (Fe, Mn and Zn), a post-transitional metal (Al), alkaline Earth metals (Ca and Mg), alkali metals (K and Na), a metalloid (Si), non-metals (P and S) and a halogen (Cl).

Transition metals (Fe, Mn, Zn and copper (Cu)) are arguably the most studied inorganic elements found in insect cuticle [9,15,24,50,51]. Given their adaptive role in the cuticle, such as increased hardness and elastic modulus, transition metals are localized or colocalized in regions of cuticular “tools” responsible for cutting or piercing through hard substrates [20,51]. Cu was not found in the cuticle of cicada mouthparts, but the transition metals Fe, Mn and Zn were present. However, Fe was only found in small abundances in the mouthparts of *M. cassinii* and *N. linnei*.

Mn and Zn were colocalized at the distal regions of the mouthparts, particularly where the mandibular bumps were found (Figure 2, Figure 4 and Figure 5; Supplementary Figure S5)—a region subjected to high friction forces and wear during the piercing mechanism. Similar colocalization patterns of Mn and Zn have been found in the cuticular tools of several other distantly related groups, including wasps [36,50,51], beetles [13], spiders [19,20,52] and polychaetes [53,54], that also are subjected to wear or breakage. As shown in this study, Zn and Mn often colocalized with the halogen Cl (Figure 6, Supplementary Figure S5).

Zn can form cross-links with nitrogen on histidine amino acids, possibly as Zn(His)_4_ or as Zn(His)_3_Cl [51,53,55] and these additional chemical bonds augment the mechanical properties of the cuticle. The role of Mn in increasing cuticular hardness is more contentious [9,50]. Recent evidence suggests that Mn not only has the capacity to perform similarly as Zn [51,54], but at concentrations lower than what is required of Zn. Mn can create a range of bonds with protein ligands (up to six), whereas Zn can only create three bonds [18,54,56]. The colocalization of Mn and Zn with Cl suggests that manganese chloride (MnCl_2_) and zinc chloride (ZnCl_2_) might be present, but Cl could be present in other compounds too, such as chlorotyrosines, that are often found in regions of cuticle with extensive sclerotization [57].

The proposed role of Zn and Mn was supported in this study, as the distal regions of the mandibles, where Zn and Mn were primarily located, were harder and had greater elastic modulus than the proximal regions (Figure 8). In addition, *N. linnei* and *M. septendecula* had harder mouthparts and relatively more Zn and Mn, further supporting their adaptive role (Figure 4 and Figure 5). Hardness is defined as a material’s resistance to permanent deformation when a particular force is applied and elastic modulus is the ratio of stress to strain during the deformation of a material [58,59]. These mechanical properties have been measured on the cuticle of a wide variety of invertebrates, including bed bugs [60], beetles [61,62], grasshoppers [44], flies [11], among others. Here, the distal region of the cicada mandibles had an average elastic modulus of 2.16 GPa and hardness of 155.05 MPa (Figure 8). These values are similar to those reported for other insect species, such as the elytra on the dung beetle, *Geotrupes stercorarius* [63,64] and the pre-stomal teeth of the yellow dung fly, *Scathophaga stercoraria* [11] and are comparable to the polymer polycarbonate [65]. The reported values here, however, might differ from those of living cicadas, because the mechanical properties of the cuticle are largely affected by the extent of hydration [66].

The deposition of Mn and Zn into the cuticle occurs chronologically where Mn is incorporated before Zn and both take place after cuticle has formed and sclerotized [8,55,67]. Given that metal incorporation occurs after the cross-linked matrix of cuticle has already formed, the mode of transportation of metal ions into the cuticle requires further study. At this point in time, the leading hypothesis for metal incorporation relates to the discovery of channels in spider fangs, up to 50 nm in diameter, that might be used for transporting Zn and Cl to specific locations [68].

The maxillae likely had lower hardness and elastic modulus values because Zn was almost entirely absent and Mn was present in lower quantities than that found in the mandibles. The sheath displayed a different pattern of elemental composition mostly by lacking transition metals and instead having larger amounts of Mg and K (Supplementary Figures S1 and S3). The contribution of Mg and K to the mechanical properties of insect cuticle, however, is not clearly understood. The hard material high-magnesium calcite ((CaMg(CO_3_)_2_) was previously reported in the exoskeletons of the leaf-cutter ant, *Acromyrmex echinatior* [69]. The lack of a correlation between Ca and Mg in cicada mouthparts indicates the absence of high-magnesium calcite. Given that the sheath does not pierce wood, the large amount of Mg suggests an adaptive role other than increased hardness or greater elastic modulus and perhaps contributes to decreasing susceptibility to fracturing.

We previously reported the elemental composition of cicada ovipositors [34], using the same individuals used in this study, thus providing an opportunity to compare two piercing structures from the same group of individuals. In the present study, the abundance of Zn was in relatively high concentrations in the mandibles where it averaged approximately 0.55%wt (1.1%wt at distal and tip regions) but was nearly absent in the ovipositors (0.02%wt). The lack of Zn in the mouthpart structures not responsible for piercing (maxillae had 0.01%wt and the sheath had 0%wt) was expected, but the lack of Zn in the ovipositors, which do pierce, indicates a potential mechanism whereby particular elements, including transition metals, are differentially allocated to specific regions on specific structures. This proposed hypothesis is further supported by examining the allocation of Mn, where the cicada ovipositors had an average of 0.2%wt of Mn at an abundance slightly higher than those reported here for the mandibles (0.09%wt for the entire mandible, 0.17%wt at the distal and tip regions). The periodical cicada, *M. cassinii*, displayed the greatest differential in Mn abundance with an average of 0.31%wt in the ovipositor compared to 0.06%wt in the distal and tip regions of the mandibles [34]. For *M. septendecim*, the pattern was the opposite where there were high levels in the tip and distal regions of the mandible (average 0.17%wt) but only small quantities in ovipositors (0.02%wt). The mouthparts of *N. linnei* had less Mn than the ovipositors and interestingly, *M. septendecula* completely lacked Mn in its ovipositor but had high amounts of it in the mandibles (0.30%wt).

Although Mn and Zn both likely contribute to hardness and elastic modulus properties, the complex nature of their chemistry and bond-formation potential with other elements that can create a variety of molecules suggests that they might be able to contribute to other mechanical properties. For example, Mn might play an important role in preventing fracturing, which would include the formation of other chemical bonds. Unfortunately, nanoindentation was not used in the ovipositor study to assess mechanical properties, which could have provided an opportunity to examine how Mn contributes to hardness or elastic modulus in a general absence of Zn.

The life history of cicadas makes them difficult to study. The periodical cicadas, for example, spend up to 17 years underground, so key aspects of their biology, such as growth rates and feeding preferences remain relatively unknown. Here, we consider element presence and abundance and how these inorganic elements are distributed to various structures as a method for determining cicada life history traits. The presence and abundance of inorganic elements in cicada cuticle likely comes from ions that are ingested while feeding on the xylem from trees. Trees host a variety of inorganic elements in their xylem [70,71] and cicadas are likely to begin acquiring these elements as immature nymphs; however, it is unknown if inorganic element acquisition begins at early stages of cicada development or closer to the adult stage. If tree species differ in the presence and abundance of elements, this could be reflected by the cicada cuticle. In addition, tree species differ in their hardness and we could expect that cicadas with harder mouthparts and ovipositors are adapted for harder trees, similar to what has been found regarding feeding behaviors of termites [72] and oviposition preferences of damselflies [73]. However, in this study, different cicada species overlapped in element abundance and presence, hence these characters were not useful for species delineation (Figure 7). In addition, although the cicada species studied here differ in their mouthpart morphology, the similarities among their structures make it difficult to use morphology as a tool to assess specific feeding preferences.

It is unclear if the absence of particular inorganic elements in structures, such as a lack of Zn in the ovipositor [34], is due to prioritizing Zn distribution to the distal regions of the mandibles or if there is a lack of a mechanism to allocate this element to the ovipositor. It is also unclear as to why natural selection has apparently favored Mn distribution in the ovipositor, but not Zn, which could be adaptive in facilitating ovipositor piercing. These questions represent some of the most compelling questions in this field: how are inorganic elements distributed to specific locations in the cuticle and what mechanism of selection is in place to ensure particular elements reach specific structures?

It is now known that adult cicadas feed, but this was not always known. The idea that cicadas do not feed dates to Plato in ancient Greece, who wrote that cicadas were originally men that were enchanted by the Muses to sing for so long that they did not eat, and died. The Muses, as a reward, turned the men into cicadas, so they could sing all day without the need to eat. The view that adult cicadas did not eat continued until Paul Dudley in 1733, corrected it writing, “some have inclined to think (cicadas) eat nothing…but at length by a careful observation, it has been found that they are nourished by the juices of the tender twigs, especially of young apple trees, which they draw out by piercing them with the proboscis” [74].

## 5. Conclusions

The present study provides additional information about how cicadas are likely to feed.

The sheath lacks significant amounts of transition metals, suggesting that its main function is to house the stylets, possibly keeping them clean from debris and injury. Once a suitable tree host is located, the stylet exits the sheath and the mandibles begin antiparallel piercing movements to reach xylem, which is facilitated by having larger abundances of transition metals in the distal regions. After the wood is pierced, the maxilla enters the vascular bundle to feed on xylem by a sucking mechanism that requires the sucking pump in the cicada’s head to induce a pressure differential. Although we now know that adult cicadas feed, several questions remain regarding the mechanism for the allocation of inorganic elements and additional studies are needed to determine how elements other than transition metals, such as K, Na, P and Si, augment the insect cuticle.

## Figures and Tables

**Table 1:** ANOVA results of inorganic element abundance in cicada mouthpart structures and locations within structures.

	*M. cassinii*						*M. septendecim*					
	Mouthpart		Location		Mouthpart × Location		Mouthpart		Location		Mouthpart × Location	
	*F*	*P*	*F*	*P*	*F*	*P*	*F*	*P*	*F*	*P*	*F*	*P*
**Al**	2.94	0.062	0.353	0.787	0.35	0.905	2.78	0.072	2.56	0.066	2.56	**0.031**
**Ca**	1.47	0.239	2.37	0.082	2.17	0.062	0.43	0.654	1.22	0.313	0.79	0.586
**Cl**	5.46	**0.007**	10.62	**<0.001**	2.95	**0.016**	3.06	0.056	3.94	**0.014**	1.26	0.292
**Fe**	1.68	0.198	1.51	0.223	0.59	0.733	**-**	**-**	**-**	**-**	**-**	**-**
**K**	6.97	**0.002**	3.18	**0.032**	2.29	0.051	0.31	0.733	2.67	0.058	0.72	0.637
**Mg**	0.87	0.426	0.56	0.646	2.44	**0.038**	4.55	**0.015**	2.58	0.065	5.72	**<0.001**
**Mn**	7.16	**0.002**	16.58	**<0.001**	3.73	**0.004**	1.43	0.248	3.33	**0.027**	0.96	0.464
**Na**	22.36	**<0.001**	13.36	**<0.001**	6.74	**<0.001**	42.93	**<0.001**	11.94	**<0.001**	9.54	**<0.001**
**P**	0.58	0.561	0.55	0.651	1.05	0.401	2.50	0.093	0.89	0.451	1.17	0.340
**S**	0.19	0.831	0.06	0.980	0.685	0.663	0.20	0.822	0.36	0.780	0.88	0.517
**Si**	0.68	0.514	0.36	0.853	0.52	0.792	0.98	0.382	0.75	0.529	0.84	0.543
**Zn**	43.21	**<0.001**	15.75	**<0.001**	14.97	**<0.001**	16.10	**<0.001**	5.97	**0.002**	5.40	**<0.001**
	*M. septendecula*						*N. linnei*					
	**Mouthpart**		**Location**		**Mouthpart ×** **Location**		**Mouthpart**		**Location**		**Mouthpart ×** **Location**	
	***F***	***P***	***F***	***P***	***F***	***P***	***F***	***P***	***F***	***P***	***F***	***P***
**Al**	**-**	**-**	**-**	**-**	**-**	**-**	0.72	0.494	0.95	0.424	1.66	0.152
**Ca**	5.23	**0.013**	6.02	**0.003**	2.06	0.097	0.57	0.572	0.67	0.573	1.27	0.291
**Cl**	2.06	0.150	0.92	0.447	0.34	0.911	0.19	0.830	0.56	0.641	1.01	0.430
**Fe**	**-**	**-**	**-**	**-**	**-**	**-**	1.16	0.323	1.28	0.291	1.16	0.346
**K**	4.16	**0.028**	2.84	0.060	0.42	0.859	0.73	0.485	0.90	0.449	1.06	0.398
**Mg**	0.01	0.994	0.15	0.930	1.45	0.237	10.53	**<0.001**	1.75	0.170	4.32	**0.001**
**Mn**	6.58	**0.005**	5.53	**0.005**	3.23	**0.018**	5.24	**0.009**	5.17	**0.004**	3.45	**0.007**
**Na**	2.79	0.081	0.21	0.888	0.17	0.983	7.23	**0.002**	8.88	**<0.001**	4.02	**0.002**
**P**	0.76	0.477	3.23	**0.040**	1.43	0.245	0.87	0.427	1.21	0.318	0.62	0.717
**S**	5.24	**0.013**	2.40	0.093	0.67	0.674	1.03	0.364	0.77	0.516	1.06	0.399
**Si**	1.99	0.159	0.16	0.923	0.58	0.941	0.27	0.767	0.154	0.215	0.27	0.948
**Zn**	13.28	**0.001**	6.60	**0.002**	6.59	**0.003**	16.06	**<0.001**	6.01	**0.001**	6.01	**<0.001**

Mouthpart = mandible, maxilla, or sheath. Location = location on mouthpart (tip, distal, mid and proximal). Significant values (*p* < 0.05) are shown in **bold**.

**Table 2:** Pearson correlations (*r*) between elements found in the mouthpart structures of cicadas.

Mandible														
	Al	C	Ca	Cl	Fe	K	Mg	Mn	Na	O	P	S	Si	Zn
Al *	**-**	**-**	**-**	**-**	**-**	**-**	**-**	**-**	**-**	**-**	**-**	**-**	**-**	**-**
**C**	**-**	**-**	−0.20	**−0.51**	0.03	−0.27	0.26	**−0.52**	−0.31	**0.85**	0.08	−0.18	**−0.40**	**−0.52**
**Ca**	**-**	**-**	**-**	0.30	−0.12	0.20	−0.17	0.38	0.14	−0.34	0.18	0.22	−0.06	**0.39**
**Cl**	**-**	**-**	**-**	**-**	0.07	**0.65**	−0.17	**0.79**	**0.73**	**−0.85**	−0.15	0.21	−0.04	**0.87**
**Fe**	**-**	**-**	**-**	**-**	**-**	−0.01	−0.06	0.03	0.14	−0.02	−0.06	−0.05	−0.04	0.05
**K**	**-**	**-**	**-**	**-**	**-**	**-**	−0.07	**0.46**	0.32	**−0.51**	−0.19	**0.46**	−0.11	0.14
**Mg**	**-**	**-**	**-**	**-**	**-**	**-**	**-**	−0.21	−0.26	**−0.63**	**0.51**	0.03	−0.09	−0.21
**Mn**	**-**	**-**	**-**	**-**	**-**	**-**	**-**	**-**	**0.60**	**−0.81**	−0.19	0.07	0.07	**0.83**
**Na**	**-**	**-**	**-**	**-**	**-**	**-**	**-**	**-**	**-**	**−0.63**	0.17	−0.07	−0.03	**0.72**
**O**	**-**	**-**	**-**	**-**	**-**	**-**	**-**	**-**	**-**	**-**	0.17	−0.16	−0.17	**−0.88**
**P**	**-**	**-**	**-**	**-**	**-**	**-**	**-**	**-**	**-**	**-**	**-**	0.15	−0.23	−0.18
**S**	**-**	**-**	**-**	**-**	**-**	**-**	**-**	**-**	**-**	**-**	**-**	**-**	−0.07	0.09
**Si**	**-**	**-**	**-**	**-**	**-**	**-**	**-**	**-**	**-**	**-**	**-**	**-**	**-**	−0.06
**Zn**	**-**	**-**	**-**	**-**	**-**	**-**	**-**	**-**	**-**	**-**	**-**	**-**	**-**	**-**
**Maxillae**														
	**Al**	**C**	**Ca**	**Cl**	**Fe**	**K**	**Mg**	**Mn**	**Na**	**O**	**P**	**S**	**Si**	**Zn**
Al *	-	-	-	-	-	-	-	-	-	-	-	-	-	-
**C**	-	-	−0.13	−0.13	−0.01	−0.13	−0.05	−0.08	0.05	0.15	−0.09	−0.15	−0.15	−0.08
**Ca**	-	-	-	**0.87**	−0.01	**1.00**	−0.06	0.16	−0.07	−0.18	0.16	**0.89**	0.04	0.23
**Cl**	-	-	-	-	−0.03	**0.85**	−0.02	**0.59**	−0.03	**−0.90**	**0.48**	**0.96**	0.07	**0.62**
**Fe**	-	-	-	-	-	−0.01	0.21	−0.05	0.17	0.03	0.11	0.01	−0.06	−0.02
**K**	-	-	-	-	-	-	−0.06	0.16	−0.08	**−0.92**	0.12	**0.87**	0.04	**0.91**
**Mg**	-	-	-	-	-	-	-	0.04	0.17	0.06	−0.13	−0.02	−0.07	−0.06
**Mn**	-	-	-	-	-	-	-	-	−0.06	−0.34	**0.71**	**0.49**	0.01	**0.91**
**Na**	-	-	-	-	-	-	-	-	-	0.07	−0.08	−0.01	−0.06	−0.04
**O**	-	-	-	-	-	-	-	-	-	-	−0.27	**−0.91**	−0.36	**−0.39**
**P**	**-**	**-**	**-**	**-**	**-**	**-**	**-**	**-**	**-**	**-**	**-**	0.15	−0.23	−0.18
**S**	**-**	**-**	**-**	**-**	**-**	**-**	**-**	**-**	**-**	**-**	**-**	**-**	−0.07	0.09
**Si**	**-**	**-**	**-**	**-**	**-**	**-**	**-**	**-**	**-**	**-**	**-**	**-**	**-**	−0.06
**Zn**	**-**	**-**	**-**	**-**	**-**	**-**	**-**	**-**	**-**	**-**	**-**	**-**	**-**	**-**
**Sheath**														
	**Al**	**C**	**Ca**	**Cl**	**Fe**	**K**	**Mg**	**Mn**	**Na**	**O**	**P**	**S**	**Si**	**Zn**
**Al**	-	−0.30	0.03	0.01	0.12	0.30	−0.10	0.30	−0.01	−0.04	−0.03	0.20	0.11	−0.02
**C**	-	-	**−0.40**	−0.36	−0.11	**−0.59**	−0.06	−0.21	−0.28	**0.82**	**−0.57**	**−0.53**	**−0.73**	0.11
**Ca**	-	-	-	0.15	0.20	**0.64**	0.04	**0.57**	−0.01	**−0.47**	0.08	0.35	0.11	−0.01
**Cl**	-	-	-	-	0.01	0.27	0.04	0.06	**0.95**	**−0.76**	0.02	**0.51**	−0.16	−0.03
**Fe**	-	-	-	-	-	0.09	−0.09	**0.67**	0.01	−0.15	−0.02	0.04	0.04	−0.02
**K**	-	-	-	-	-	-	**0.51**	0.34	0.14	**−0.59**	**0.39**	**0.60**	0.12	−0.14
**Mg**	-	-	-	-	-	-	-	0.13	−0.12	−0.29	−0.08	0.03	−0.03	−0.08
**Mn**	-	-	-	-	-	-	-	-	−0.03	−0.29	−0.38	0.22	0.02	−0.04
**Na**	-	-	-	-	-	-	-	-	-	**−0.69**	−0.01	**0.50**	−0.22	−0.01
**O**	-	-	-	-	-	-	-	-	-	-	−0.38	**−0.65**	−0.34	0.10
**P**	-	-	-	-	-	-	-	-	-	-	-	0.31	0.30	−0.01
**S**	-	-	-	-	-	-	-	-	-	-	-	-	0.01	−0.10
**Si**	-	-	-	-	-	-	-	-	-	-	-	-	-	−0.10
**Zn**	-	-	-	-	-	-	-	-	-	-	-	-	-	-

* Aluminum was not detected in the mandibles and maxillae. Significant correlations (*p* < 0.05) are recorded in **bold.**

**Table 3:** Linear discriminant analyses for cicada mouthpart morphology and inorganic element cuticle composition.

Morphology				
Species	*M. cassinii*	*M. septendecim*	*M. septendecula*	*N. linnei*
*M. cassinii*	3	0	0	0
*M. septendecim*	0	5	0	0
*M. septendecula*	0	0	3	0
*N. linnei*	0	0	0	5
**Inorganic elements**				
**species**	***M. cassinii***	***M. septendecim***	***M. septendecula***	***N. linnei***
*M. cassinii*	3	1	0	1
*M. septendecim*	2	3	0	0
*M. septendecula*	0	0	3	0
*N. linnei*	1	0	1	3

## Data Availability

The datasets used and/or analysed during the current study are available from the corresponding author on reasonable request.

## Supplementary Materials

The following supporting information can be downloaded at: https://www.mdpi.com/article/10.3390/biology12020207/s1, Figure S1: Violin plots showing differences in potassium (K) abundance in mouthparts and locations within the mouthparts of *Magicicada cassinii*; Figure S2: Violin plots demonstrating differences in sodium (Na) abundance at different locations of cicada mouthpart structures; Figure S3: Violin plots showing differences in the abundance of magnesium (Mg) in mouthpart structures and locations within the structures of *Neotibicen linnei*; Figure S4: Violin plots showing differences in aluminum (Al) abundance in mouthparts and locations within the mouthparts of *Magicicada septendecim*; Figure S5: Principal component analysis (PCA) of all four cicada species; Table S1: Morphological measurements (mean ± S.E.) of cicada mouthpart structures; Table S2: ANOVA results of measurements of mouthpart morphology among cicada species; Table S3: ANOVA results of width measurements of mouthpart structures within cicada species; Table S4: Measurements (mean ± S.E.) of bump number and sizes among different cicada species; Table S5: ANOVA results of measurements of mandibular bumps among cicada species; Table S6: MANOVA results of differences among inorganic element abundance in mouthpart structure, location on each structure, cicada species and the interactions of these variables; Table S7: ANOVA results analyzing differences of inorganic element abundance in mouthparts (M), locations on mouthparts (L), cicada species (S) and the interactions of these variables; Table S8: Pearson’s correlations (*r*) between elements in *M. cassinii*; Table S9: Pearson’s correlations (*r*) between elements in *M. septendecim*; Table S10: Pearson’s correlations (*r*) between elements in *M. septendecula*; Table S11: Pearson’s correlations (*r*) between elements in *N. linnei*; Table S12: Explained variance of each of the principal components analyzed for mouthparts; Table S13: Table of loadings of all variables for each of the first three principal components studied for cicada mouthparts; Table S14: Explained variance of each of the principal components analyzed for locations within mouthparts; Table S15: Table of loadings of all variables for each of the first three principal components studied for location on cicada mouthparts; Table S16: ANOVA results comparing hardness (H) and elastic modulus (EM) between proximal and distal locations on the mandibles within cicada species; Table S17: ANOVA results comparing hardness (H) and elastic modulus (EM) between proximal and distal locations on the mandibles among cicada species.
