# Supplementary material for: Comparative Material and Mechanical Properties among Cicada Mouthparts: Cuticle Enhanced with Inorganic Elements Facilitates Piercing through Woody Stems for Feeding
Source: Biology (Basel). 2023 Jan 29;12(2):207. doi: 10.3390/biology12020207 (PMC9953083; doi:10.3390/biology12020207)
Supplement: Supplementary file 1 [file biology-12-00207-s001.zip › biology-2150076-supplementary.pdf]

SUPPLEMENTARY MATERIALS

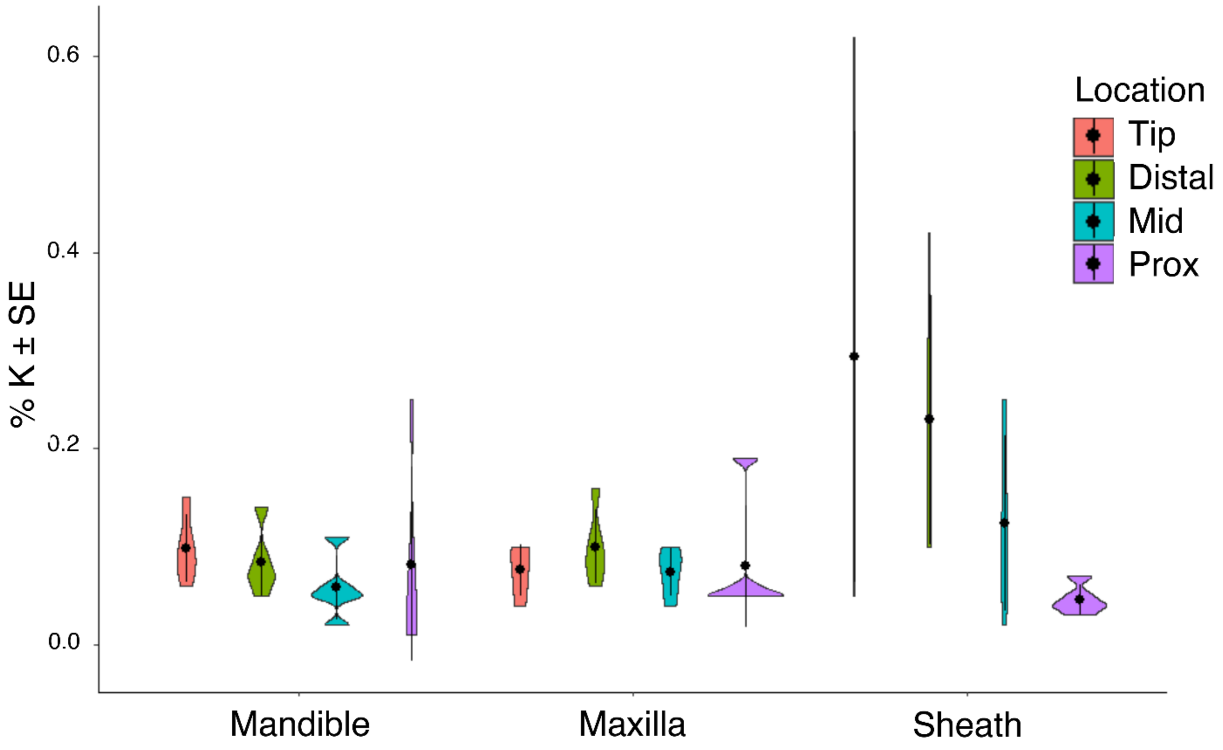

Figure S1. Violin plots showing differences in potassium (K) abundance in mouthparts and locations within the mouthparts of *Magicicada cassinii*. Center points in each violin represent the mean, with standard error bars.

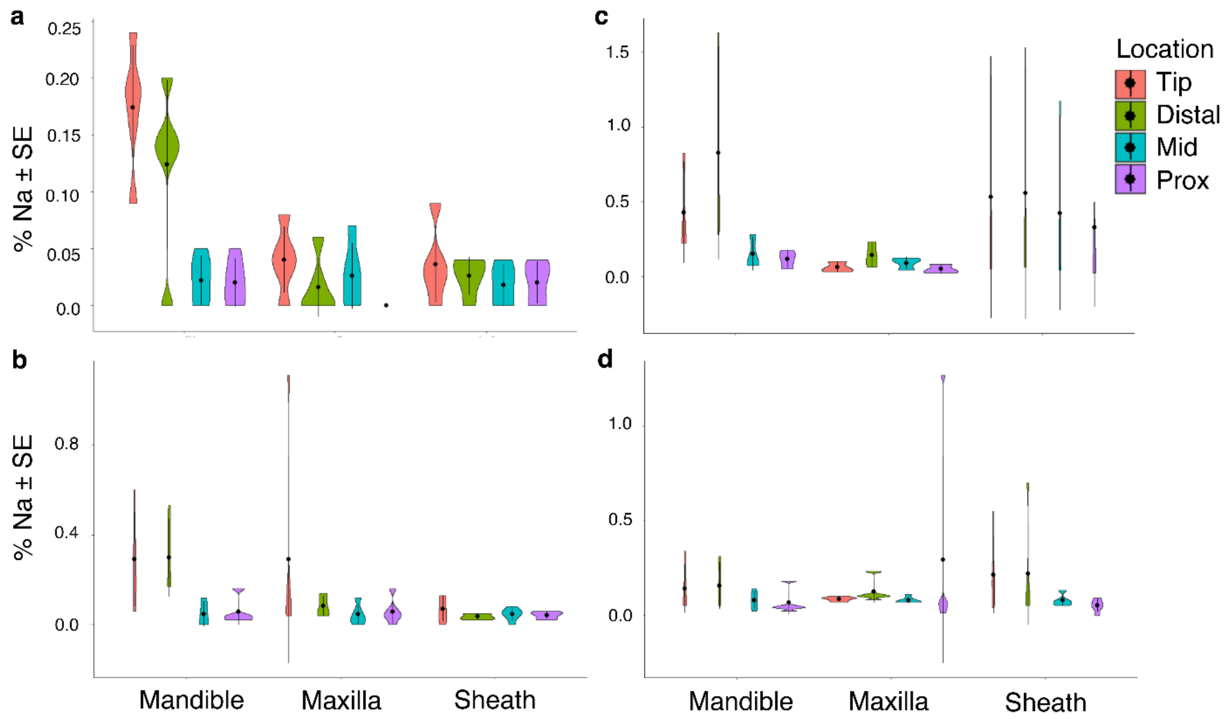

Figure S2. Violin plots demonstrating differences in sodium (Na) abundance at different locations of cicada mouthpart structures. (a) *M. cassinii*, (b) *M. septendecim*, (c) *M. septendecula*, and (d) *N. linnei*. Center points in each violin represent the mean, with standard error bars.

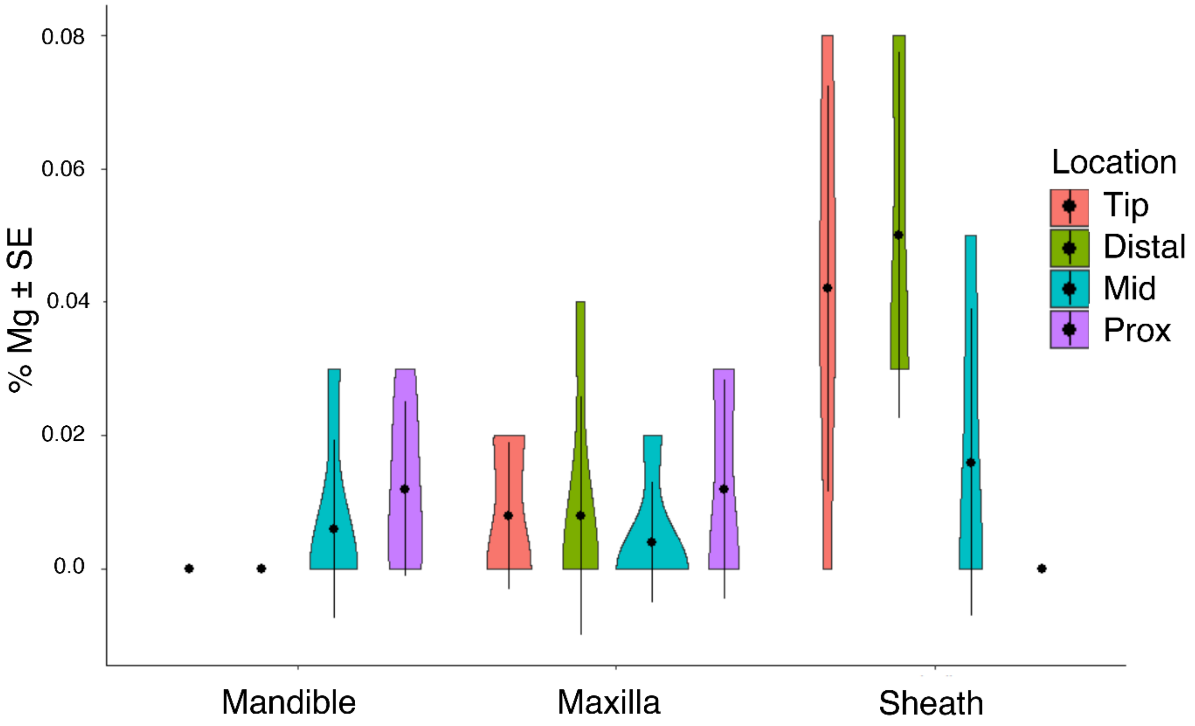

Figure S3. Violin plots showing differences in the abundance of magnesium (Mg) in mouthpart stuctures and locations within the structures of *Neotibicen linnei*. Center points in each violin represent the mean, with standard error bars.

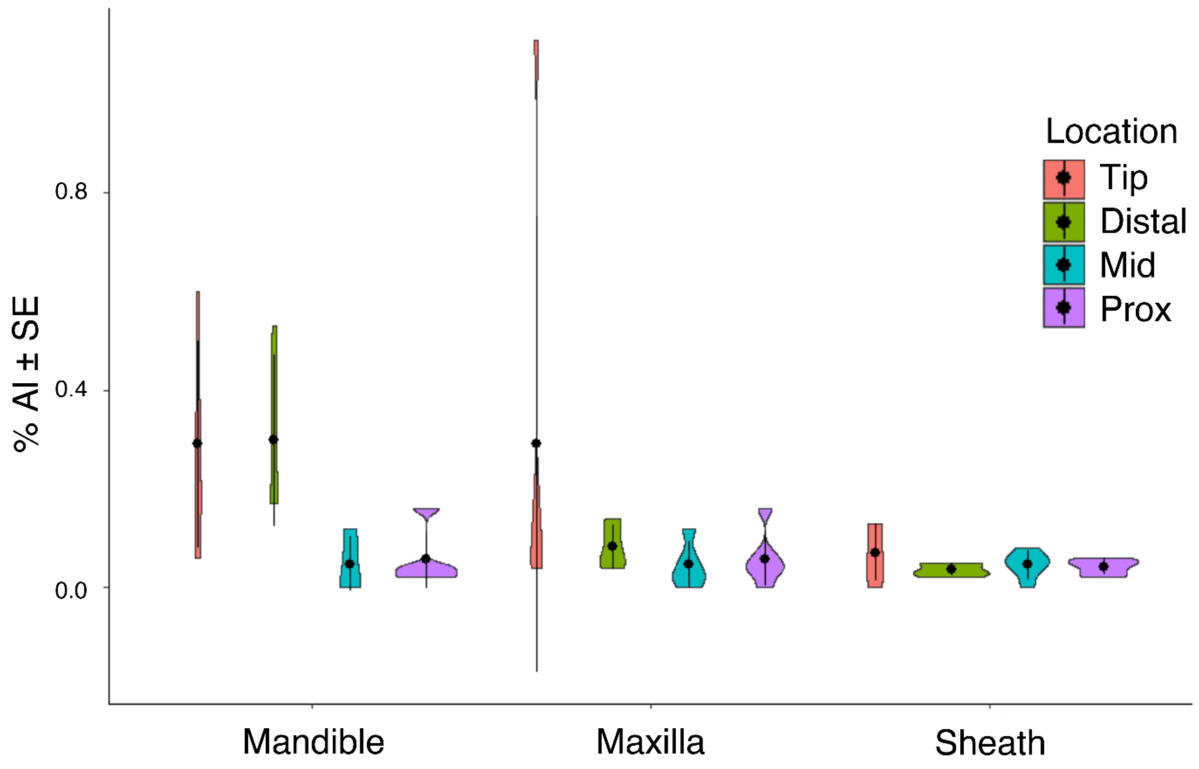

Figure S4. Violin plots showing differences in aluminum (Al) abundance in mouthparts and locations within the mouthparts of *Magicicada septendecim*. Center points in each violin represent the mean, with standard error bars.

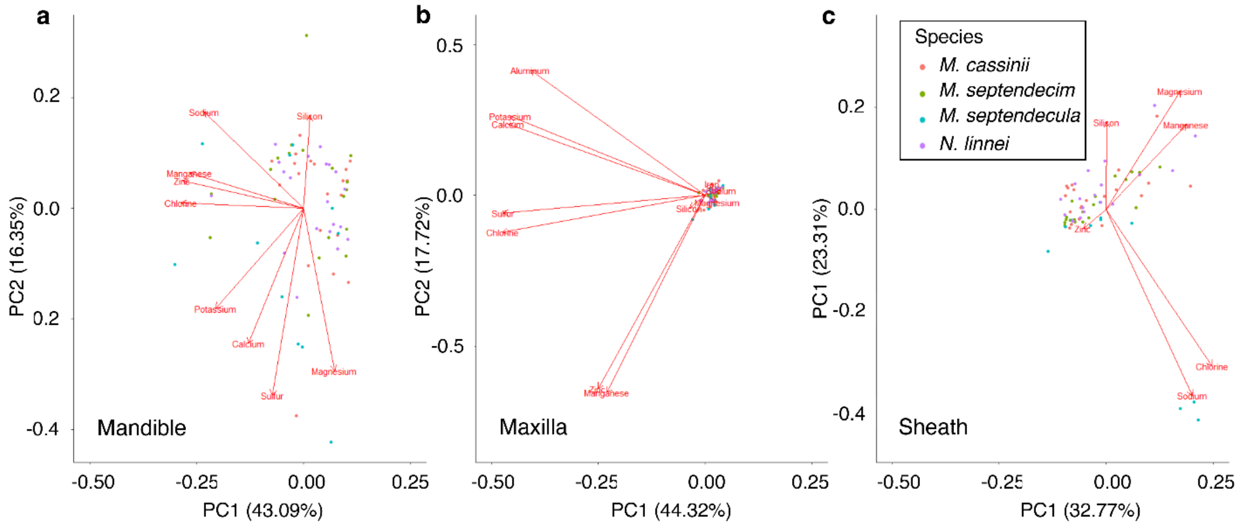

Figure S5. Principal component analysis (PCA) of all four cicada species. Plots show the variation in abundance of inorganics elements in the (a) mandibles, (b) maxillae, and (c) sheath. Individual species are represented by different colors in the ordination.

Table S1. Morphological measurements (mean±S.E.) of cicada mouthpart structures.

Species	Structure	n	Length (μm)	Location	Width (μm)
M. cassinii	Mandible	5	4235.28±74.93**A**	1	53.51±6.57**B**
				2	55.77±5.01**C**
				3	60.14±3.50**C**
	Maxillae	5	4356.93±117.75**A**	1	39.58±1.18**C** (4)
				2	43.23±3.38**B** (4)
				3	45.63±1.34**B** (3)
	Sheath	5	3732.05±63.55**A**	1	183.04±7.57**C**
				2	185.72±7.42**C**
				3	201.05±7.23**B**
M. septendecim	Mandible	5	5106.21±199.23**B**	1	62.97±2.36**AB, b**
				2	66.40±1.20**BC, ab**
				3	73.44±3.43**BC, a**
	Maxillae	5	4759.56±141.05**B**	1	46.29±1.24**C, a**
				2	46.38±1.26**B, a**
				3	50.08±1.46**B, a**
	Sheath	5	4089.52±108.84**B**	1	214.76±4.26**BC, b**
				2	219.55±5.83**BC, ab**
				3	233.23±6.81**B, a**
M. septendecula	Mandible	3	5237.64±223.64**B**	1	57.29±4.82**AB, a**
				2	58.19±2.35**B, a**
				3	64.11±4.50**AB, a**
	Maxillae	3	4416.05±611.29**B**	1	40.13±1.38**B, a**
				2	42.53±0.14**B, b**
				3	46.36±0.19**B, b**
	Sheath	3	4569.63±657.44**BC**	1	197.34±15.39**B, a**
				2	207.78±7.48**B, a**
				3	215.52±28.76**B, a**
N. linnei	Mandible	5	7036.53±206.62**C**	1	73.07±4.21**A, a**
				2	79.93±3.33**A, a**
				3	80.17±5.81**A, a**
	Maxillae	5	6678.13±153.32**B**	1	51.66±1.21**A, a**
				2	54.72±1.20**A, b**
				3	58.87±1.52**A, b**
	Sheath	5	5509.43±107.68**C**	1	244.64±11.23**A, a**
				2	257.18±9.80**A, b**
				3	294.60±8.08**A, b**

Bold capital letters indicate significant differences ($p < 0.05$) and rankings per structure among species.
Bold lowercase letters indicate significant differences within locations for each structure among species.

Table S2. ANOVA results of measurements of mouthpart morphology among cicada species.

Structure	Location	df	F	p-value
Mandible	Length	3,14	46.8360	**< 0.0001**
	Width (Location 1)	3,14	3.3166	0.0511
	Width (Location 2)	3,14	9.9606	**0.0009**
	Width (Location 3)	3,14	4.2626	**0.0246**
Maxilla	Length	3,14	23.6768	**< 0.0001**
	Width (Location 1)	3,13	20.5719	**< 0.0001**
	Width (Location 2)	3,13	9.0962	**0.0017**
	Width (Location 3)	3,12	18.5021	**< 0.0001**
Sheath	Length	3,14	13.1823	**0.0002**
	Width (Location 1)	3,14	8.7308	**0.0016**
	Width (Location 2)	3,14	15.2654	**0.0001**
	Width (Location 3)	3,14	13.8561	**0.0002**

p-values in **bold** represent significant differences among lengths and widths among mouthpart structures.

Table S3. ANOVA results of width measurements of mouthpart structures within cicada species

Species	Structure	df	F	p-value
M. cassinii	Mandible	2,12	0.4238	0.6640
	Maxilla	2,8	1.5951	0.2612
	Sheath	2,12	1.7200	0.2204
M. septendecim	Mandible	2,12	4.5511	**0.0338**
	Maxilla	2,12	2.6676	0.1100
	Sheath	2,12	2.8013	0.1004
M. septendecula	Mandible	2,6	0.8415	0.4763
	Maxilla	2,6	15.0477	**0.0046**
	Sheath	2,6	0.2230	0.8065
N. linnei	Mandible	2,12	0.7804	0.4801
	Maxilla	2,12	7.5301	**0.0076**
	Sheath	2,12	7.0541	0.0094

p-values in **bold** represent significant differences among widths within structures

Table S4. Measurements (mean±S.E.) of bump number and sizes among different cicada species.

Species	n	Bump number	Length (μm)	Width (μm)
M. cassinii	1	2**B**	22.68	3.71
M. septendecim	4	6.00±0.38**A**	29.01±2.90	6.79±0.62
M. septendecula	2	6.00**A**	30.14±7.99	5.54±0.22
N. linnei	2	1.50±0.50**B**	40.64±7.69	4.81±0.74

Bolded capital letters indicate significant differences ($p < 0.05$) and rankings per structure among species.

Table S5. ANOVA results of measurements of mandibular bumps among cicada species.

	df	F	p-value
Bump Number	3,5	25.1481	**0.0019**
Bump width	3,4	1.2015	0.4162
Bump length	3,4	3.4743	0.1301

p-values in **bold** represent significant differences among species.

Table S6. MANOVA results of differences among inorganic element abundance in mouthpart structure, location on each structure, cicada species, and the interactions of these variables.

	Wilks' λ	*F*	Num *df*	Den *df*	*P*
Mouthpart structure	0.83	7.27	30	310	**<0.001**
Location	0.71	3.22	45	468	**<0.001**
Species	0.70	3.19	45	468	**<0.001**
Mouthpart × location	1.24	2.76	90	954	**<0.001**
Mouthpart × species	1.09	2.34	90	954	**<0.001**
Location × species	1.05	1.43	135	1458	**<0.001**
Mouthpart × location × species	1.76	1.76	270	2520	**0.006**

Mouthpart structure = mandible, maxilla, or sheath. Location = location on mouthpart structure (tip, distal, mid, and proximal). Species = species of cicada (*M. cassinii*, *M. septendecim*, *M septendecula*, or *N. linnei*). Significant values are in **bold**.

Table S7. ANOVA results analyzing differences of inorganic element abundance in mouthparts (M), locations on mouthparts (L), cicada species (S), and the interactions of these variables.

	**M**		**L**		**S**		**M x L**		**M x S**		**L x S**		**M x L x S**	
	F	***P***	***F***	***P***	***F***	***P***	***F***	***P***	***F***	***P***	***F***	***P***	***F***	***P***
**Al**	3.21	**0.043**	2.83	**0.040**	1.64	0.181	3.33	**0.004**	1.97	0.073	1.85	0.062	1.79	**0.030**
**C**	1.48	0.230	0.86	0.463	0.88	0.453	1.26	0.279	0.56	0.76	0.98	0.461	0.93	0.547
**Ca**	0.59	0.558	0.67	0.570	1.54	0.207	1.07	0.380	0.48	0.823	0.64	0.764	1.10	0.354
**Cl**	1.55	0.216	4.13	**0.007**	7.81	**<0.001**	1.18	0.319	3.19	**0.005**	0.96	0.498	0.63	0.873
**Fe**	1.58	0.209	1.65	0.180	0.91	0.439	1.29	0.263	0.85	0.534	0.96	0.472	0.91	0.570
**K**	0.45	0.637	0.36	0.783	1.01	0.389	1.11	0.360	0.81	0.562	1.10	0.363	0.87	0.619
**Mg**	8.22	**<0.001**	2.01	0.114	3.53	**0.016**	**7.75**	**<0.001**	2.04	0.062	0.57	0.817	1.48	0.102
**Mn**	12.19	**<0.001**	14.90	**<0.001**	2.76	**0.044**	6.18	**<0.001**	2.10	0.056	1.80	0.072	1.40	0.139
**Na**	7.08	**0.001**	3.96	**0.009**	9.59	**<0.001**	2.47	**0.025**	5.61	**<0.001**	0.15	1.000	0.121	1.000
**P**	0.05	0.953	0.78	0.507	1.74	0.162	1.37	0.229	1.04	0.401	0.66	0.743	0.86	0.622
**O**	7.96	**<0.001**	3.66	**0.014**	0.99	0.400	4.51	**<0.001**	2.26	**0.040**	1.48	0.160	1.04	0.423
**S**	0.51	0.604	0.69	0.559	1.88	0.134	1.29	0.264	1.26	0.280	0.75	0.663	0.74	0.76
**Si**	1.26	0.286	0.901	0.442	2.06	0.107	0.78	0.585	0.71	0.641	0.52	0.858	0.73	0.78
**Zn**	59.62	**<0.001**	22.19	**<0.001**	6.25	**<0.001**	21.75	**<0.001**	6.22	**<0.001**	4.05	**<0.001**	4.01	**<0.001**

Mouthpart = mandible, maxilla, or sheath. Location = location on mouthpart (tip, distal, mid, and proximal). Species = species of cicada (*M. cassinii*, *M. septendecim, M septendecula,* or *N. linnei*). Significant values are in **bold**.

Table S8. Pearson's correlations (*r*) between elements in *M. cassinii*.

	Al	C	Ca	Cl	Fe	K	Mg	Mn	Na	O	P	S	Si	Zn
Al	-	0.08	0.02	-0.12	0.05	0.12	0.08	-0.14	-0.05	0.09	-0.01	-0.05	-0.08	-0.09
C	-	-	-0.19	-0.33	-0.11	-0.29	0.23	-0.22	-0.23	**0.93**	-0.38	-0.22	**-0.90**	-0.27
Ca	-	-	-	0.30	-0.16	**0.69**	**0.58**	0.02	0.05	-0.25	0.38	0.32	-0.02	-0.11
Cl	-	-	-	-	0.06	0.38	0.02	**0.56**	**0.73**	-0.33	0.22	0.01	0.10	**0.68**
Fe	-	-	-	-	-	-0.08	0.05	0.05	0.06	-0.11	-0.01	0.04	0.10	0.10
K	-	-	-	-	-	-	0.39	-0.01	0.09	**-0.52**	**0.53**	0.10	0.10	-0.07
Mg	-	-	-	-	-	-	-	-0.07	**0.42**	0.17	0.01	0.18	-0.30	-0.21
Mn	-	-	-	-	-	-	-	-	0.42	-0.40	-0.15	-0.09	0.11	**0.53**
Na	-	-	-	-	-	-	-	-	-	**-0.52**	0.09	0.18	-0.06	**0.90**
O	-	-	-	-	-	-	-	-	-	-	-0.33	-0.25	**-0.73**	**-0.57**
P	-	-	-	-	-	-	-	-	-	-	-	**0.45**	0.10	-0.07
S	-	-	-	-	-	-	-	-	-	-	-	-	-0.03	0.04
Si	-	-	-	-	-	-	-	-	-	-	-	-	-	0.05
Zn	-	-	-	-	-	-	-	-	-	-	-	-	-	-

Significant correlations ($p < 0.05$) are recorded in **bold.**

Table S9. Pearson's correlations (*r*) between elements in *M. septendecim*.

	Al	C	Ca	Cl	Fe	K	Mg	Mn	Na	O	P	S	Si	Zn
Al	-	-0.12	0.01	0.01	-	0.22	-0.04	-0.01	0.06	0.04	-0.07	0.07	-0.02	-0.07
C	-	-	**-0.47**	**-0.48**	-	**-0.47**	0.10	**-0.51**	-0.17	**0.86**	-0.40	**-0.51**	**-0.42**	-0.28
Ca	-	-	-	**0.76**	-	**0.93**	0.01	**0.83**	-0.16	**-0.60**	**0.76**	**0.89**	-0.01	0.13
Cl	-	-	-	-	-	**0.77**	-0.18	**0.93**	**0.43**	**-0.78**	**0.52**	**0.69**	0.02	**0.61**
Fe*	-	-	-	-	-	-	-	-	-	-	-	-	-	-
K	-	-	-	-	-	-	0.10	**0.79**	0.03	**-0.60**	**0.66**	**0.84**	-0.03	0.19
Mg	-	-	-	-	-	-	-	-0.14	-0.16	0.12	-0.07	0.03	-0.06	-0.15
Mn	-	-	-	-	-	-	-	-	0.25	**-0.78**	**0.53**	**0.67**	0.15	**0.54**
Na	-	-	-	-	-	-	-	-	-	-0.40	-0.18	-0.01	0.11	**0.65**
O	-	-	-	-	-	-	-	-	-	-	-0.40	**-0.55**	-0.28	**-0.66**
P	-	-	-	-	-	-	-	-	-	-	-	**0.78**	-0.03	-0.07
S	-	-	-	-	-	-	-	-	-	-	-	-	-0.02	0.04
Si	-	-	-	-	-	-	-	-	-	-	-	-	-	0.01
Zn	-	-	-	-	-	-	-	-	-	-	-	-	-	-

*Iron (Fe) was not detected in *M. septendecim*. Significant correlations ($p < 0.05$) are recorded in **bold.**

Table S10. Pearson's correlations (*r*) between elements in *M. septendecula*.

	Al	C	Ca	Cl	Fe	K	Mg	Mn	Na	O	P	S	Si	Zn
Al*	-	-	-	-	-	-	-	-	-	-	-	-	-	-
C	-	-	**-0.56**	**-0.76**	-	**-0.64**	0.10	**-0.87**	-0.46	**0.99**	0.22	-0.37	-0.41	**-0.91**
Ca	-	-	-	0.17	-	0.23	0.41	**0.60**	-0.03	**-0.53**	0.09	0.20	0.17	**0.63**
Cl	-	-	-	-	-	0.48	-0.16	0.47	**0.89**	**-0.79**	-0.30	0.37	0.18	0.48
Fe	-	-	-	-	-	-	-	-	-	-	-	-	-	-
K	-	-	-	-	-	-	-0.04	0.52	0.20	**-0.61**	-0.16	**0.58**	0.23	**0.94**
Mg	-	-	-	-	-	-	-	-0.07	-0.18	**-0.87**	**0.55**	0.05	-0.02	-0.13
Mn	-	-	-	-	-	-	-	-	0.09	**-0.87**	-0.26	0.07	0.42	**0.94**
Na	-	-	-	-	-	-	-	-	-	-0.51	-0.29	0.31	-0.04	0.14
O	-	-	-	-	-	-	-	-	-	-	0.27	-0.34	-0.32	**-0.91**
P	-	-	-	-	-	-	-	-	-	-	-	-0.06	-0.03	-0.21
S	-	-	-	-	-	-	-	-	-	-	-	-	0.20	0.14
Si	-	-	-	-	-	-	-	-	-	-	-	-	-	0.33
Zn	-	-	-	-	-	-	-	-	-	-	-	-	-	-

*Aluminum (Al) and iron (Fe) were not detected in *M. septendecula* specimens. Significant correlations ($p < 0.05$) are recorded in **bold.**

Table S11. Pearson's correlations (*r*) between elements in *N. linnei*.

	Al	C	Ca	Cl	Fe	K	Mg	Mn	Na	O	P	S	Si	Zn
Al	-	-0.12	**0.87**	**0.72**	**0.49**	**0.86**	-0.05	0.03	0.01	**-0.80**	-0.05	**0.84**	-0.01	-0.07
C	-	-	-0.13	-0.13	-0.02	-0.13	-0.07	-0.08	-0.04	0.16	-0.02	-0.14	-0.15	-0.06
Ca	-	-	-	**0.85**	0.04	**0.98**	0.09	-0.03	-0.01	**-0.91**	-0.05	**0.96**	0.04	-0.05
Cl	-	-	-	-	0.04	**0.83**	0.23	0.18	0.22	**-0.87**	-0.11	-0.07	-0.10	0.14
Fe	-	-	-	-	-	0.01	-0.07	0.16	0.16	-0.05	-0.03	0.02	-0.02	-0.04
K	-	-	-	-	-	-	-0.02	-0.05	-0.07	**-0.91**	-0.04	**0.96**	0.04	-0.04
Mg	-	-	-	-	-	-	-	-0.18	0.01	0.04	-0.02	-0.04	-0.01	-0.21
Mn	-	-	-	-	-	-	-	-	**0.50**	-0.25	-0.05	0.02	0.05	**0.90**
Na	-	-	-	-	-	-	-	-	-	-0.14	0.11	0.01	-0.09	**0.63**
O	-	-	-	-	-	-	-	-	-	-	0.10	**-0.92**	-0.31	-0.22
P	-	-	-	-	-	-	-	-	-	-	-	-0.07	-0.10	-0.08
S	-	-	-	-	-	-	-	-	-	-	-	-	0.10	-0.01
Si	-	-	-	-	-	-	-	-	-	-	-	-	-	-0.06
Zn	-	-	-	-	-	-	-	-	-	-	-	-	-	-

Significant correlations ($p < 0.05$) are recorded in **bold.**

Table S12. Explained variance of each of the principal components analyzed for mouthparts.

Structure		Standard deviation	Proportion of variance	Cumulative proportion
Mandible	PC1	1.97	0.43	0.43
	PC2	1.22	0.16	0.59
	PC3	1.04	0.12	0.71
	PC4	0.99	0.11	0.82
	PC5	0.80	0.07	0.89
	PC6	0.63	0.04	0.94
	PC7	0.55	0.03	0.97
Maxilla	PC1	2.21	0.44	0.44
	PC2	1.40	0.18	0.62
	PC3	1.18	0.13	0.75
	PC4	0.99	0.09	0.83
	PC5	0.92	0.08	0.91
	PC6	0.89	0.07	0.98
	PC7	0.31	0.01	0.99
Sheath	PC1	1.72	0.33	0.33
	PC2	1.45	0.23	0.56
	PC3	1.04	0.12	0.68
	PC4	0.98	0.11	0.79
	PC5	0.92	0.10	0.88
	PC6	0.78	0.07	0.95
	PC7	0.48	0.26	0.98

Table S13. Table of loadings of all variables for each of the first three principal components studied for cicada mouthparts.

Structure	Element	PC1	PC2	PC3
Mandible	Al*	-	-	-
	Ca	-0.22	**-0.41**	**-0.48**
	Cl	**-0.48**	0.02	0.02
	Fe*	**-**	-	-
	K	-0.34	-0.30	0.38
	Mg	0.12	**-0.49**	**-0.53**
	Mn	**-0.45**	0.12	-0.14
	Na	**-0.40**	0.29	-0.10
	P*	**-**	-	-
	S	-0.12	**-0.56**	**0.53**
	Si	0.03	0.28	0.04
	Zn	**-0.47**	0.08	-0.13
Maxilla	Al	-0.38	0.39	-0.01
	Ca	**-0.43**	0.22	0.01
	Cl	**-0.44**	0.11	0.04
	Fe	0.02	0.04	**0.57**
	K	**-0.42**	0.24	0.01
	Mg	0.03	-0.02	**0.58**
	Mn	-0.21	**-0.61**	0.03
	Na	0.03	0.02	**0.53**
	P*	-	-	**-**
	S	**-0.44**	-0.05	0.05
	Si	-0.03	-0.04	-0.25
	Zn	-0.23	**-0.60**	0.01
Sheath	Al*	**-**	**-**	**-**
	Ca	**0.43**	**0.36**	-0.14
	Cl	**0.38**	**-0.47**	-0.01
	Fe*	**-**	**-**	**-**
	K	**0.48**	0.21	0.07
	Mg	0.27	**0.35**	-0.25
	Mn	0.29	0.25	-0.15
	Na	0.31	**-0.56**	0.01
	P	-	**-**	-
	S	**0.44**	-0.17	0.19
	Si	0.01	0.26	**0.62**
	Zn	-0.08	-0.06	**-0.69**

*Indicates metals that were not detected in specific mouthpart structures.

Table S14. Explained variance of each of the principal components analyzed for locations within mouthparts.

Location		Standard deviation	Proportion of variance	Cumulative proportion
Tip	PC1	2.01	0.34	0.34
	PC2	1.47	0.18	.52
	PC3	1.13	0.11	0.62
	PC4	1.06	0.09	0.72
	PC5	0.10	0.08	0.80
	PC6	0.94	0.07	0.87
	PC7	0.90	0.07	0.94
Distal	PC1	1.89	0.36	0.36
	PC2	1.3	0.19	0.55
	PC3	1.09	0.12	0.67
	PC4	1.00	0.10	0.77
	PC5	0.96	0.09	0.86
	PC6	0.75	0.06	0.91
	PC7	0.61	0.04	0.95
Mid	PC1	1.52	0.19	0.19
	PC2	1.35	0.15	0.35
	PC3	1.23	0.13	0.47
	PC4	1.17	0.11	0.59
	PC5	1.03	0.09	0.67
	PC6	1.01	0.09	0.76
	PC7	0.96	0.08	0.84
Proximal	PC1	2.15	0.42	0.42
	PC2	1.34	0.16	0.58
	PC3	1.13	0.12	0.70
	PC4	1.04	0.10	0.80
	PC5	1.00	0.09	0.89
	PC6	0.3	0.63	0.95
	PC7	0.63	0.4	0.99

Table S15. Table of loadings of all variables for each of the first three principal components studied for location on cicada mouthparts.

Location	Element	PC1	PC2	PC3
Tip	Al	-0.03	-0.14	0.41
	Ca	**-0.41**	-0.25	-0.24
	Cl	**-0.37**	0.36	-0.16
	Fe*	**-**	-	-
	K	**-0.47**	-0.17	0.01
	Mg	-0.05	-0.35	**-0.65**
	Mn	**-0.40**	0.17	0.12
	Na	-0.10	**0.52**	-0.17
	P*	-	**-**	-
	S	**-0.47**	-0.11	0.05
	Si	-0.12	-0.16	**0.38**
	Zn	-0.11	**0.53**	0.03
Distal	Al	-0.01	-0.35	-0.37
	Ca	-0.20	**-0.45**	**0.46**
	Cl	**-0.46**	0.10	-0.15
	Fe*	-	-	**-**
	K	**-0.36**	**-0.36**	-0.08
	Mg	0.02	**-0.63**	0.05
	Mn	**-0.40**	0.17	**0.42**
	Na	-0.35	0.23	**-0.42**
	P*	-	-	**-**
	S	**-0.39**	-0.11	-0.34
	Si	0.02	0.14	-0.25
	Zn	**-0.43**	0.16	0.30
Mid	Al	0.03	-0.10	0.01
	Ca	0.10	-0.07	-0.14
	Cl	**-0.55**	-0.14	-0.34
	Fe	0.10	0.25	-0.34
	K	**-0.39**	-0.12	**0.41**
	Mg	0.11	-0.27	-0.10
	Mn	-0.15	-0.08	**0.45**
	Na	**-0.49**	-0.15	**-0.48**
	P	-0.01	**0.60**	-0.22
	S	**-0.40**	0.34	0.18
	Si	0.20	0.27	-0.06
	Zn	-0.22	**0.48**	0.23
Proximal	Al	**0.45**	0.04	0.17
	Ca	**0.45**	0.06	-0.17
	Cl	**0.42**	0.01	**0.38**
	Fe	-0.02	-0.08	0.06
	K	**0.45**	0.04	-0.16
	Mg	-0.04	**0.64**	0.01
	Mn	-0.02	-0.05	-0.07
	Na	0.08	-0.02	**0.86**
	P	-0.05	**0.62**	-0.04
	S	**0.45**	0.02	0.03
	Si	0.05	**-0.43**	-0.13
	Zn*	-	-	**-**

Table S16. ANOVA results comparing hardness (H) and elastic modulus (EM) between proximal and distal locations on the mandibles within cicada species.

Species		df	F	p-value
M. cassinii	EM	1,16	49.6195	< 0.0001
	H	1,16	33.4714	< 0.0001
M. septendecula	EM	1,16	9.2683	0.0077
	H	1,16	8.8103	0.0091
N. linnei	EM	1,16	7.5846	0.0141
	H	1,16	9.7597	0.0065

Table S17. ANOVA results comparing hardness (H) and elastic modulus (EM) between proximal and distal locations on the mandibles among cicada species.

Location		df	F	p-value
Distal	EM	2,24	10.6273	0.0005
	H	2,24	10.0166	0.0007
Proximal	EM	2,24	1.3808	0.2706
	H	2,24	2.2683	0.1252
